# Weight-Based Standardized Sugammadex Dosing in Pediatrics: A Quality Improvement Initiative to Improve Compliance with Dosing Guidelines and Reduce Waste and Cost

**DOI:** 10.1155/2024/6049114

**Published:** 2024-08-24

**Authors:** Sydney E. S. Brown, Michael Meyer, Andrea Meyer, Ruth Cassidy, Xinyi Zhao, Deborah Wagner, Laura Wetzel, Douglas A. Colquhoun

**Affiliations:** ^1^ Department of Anesthesiology University of Michigan, Ann Arbor, MI, USA; ^2^ Division of Pediatric Anesthesiology Department of Anesthesiology University of Michigan, Ann Arbor, MI, USA

## Abstract

**Methods:**

Sugammadex vials were fractionated into 25, 50, or 100 mg aliquots, which would be distributed to anesthesia staff by pharmacy staff in approximate 2 mg/kg of actual body weight doses (±10%). We analyzed changes in sugammadex waste and dosing practices 1/1/2019 to 3/15/2023 pre/postintervention (4/1/2021). We gauged dose appropriateness using last train of four (TOF) prior to sugammadex administration.

**Results:**

7,889 patients 2–17 years (4,771 with documented TOF), ASA 1–4 receiving general anesthesia with a steroidal NMB medication and sugammadex reversal. Pre- and postintervention mean doses were 2.5 mg/kg (SD: 1.2) and 2.4 mg/kg (SD: 0.96), respectively. A smaller proportion of cases received standard 2 or 4 mg/kg doses (pre: 77.6 vs. post: 66.7%). Mean waste per case declined from 4.2 mg/kg (SD: 4.1) to 0.22 mg/kg (SD: 0.38). Among cases with 0 or 1 measured twitches on TOF that should receive at least 4 mg/kg, fewer received at least 3.6 mg/kg (post: 56.7% vs. pre: 66.8%), and a greater proportion received less than 2.2 mg/kg (post: 27.4% vs. pre: 20.7%). Among cases that should have received at least 2 mg/kg by TOF, the proportion of patients receiving more than 3.6 mg/kg declined from 9.5% to 5.2%. *Discussion*. Fractionating sugammadex vials was associated with decreases in waste, but not dose, and significant underdosing was more likely to occur. While vial fractionation could enable increased access to sugammadex and other costly medications, it may introduce unintended consequences.

## 1. Introduction

Sugammadex is a neuromuscular blockade reversal agent widely adopted in adult and pediatric anesthesia practice following its approval for adult patients in the U.S. in 2015 [1, 2]. It provides reversal of rocuronium or vecuronium-induced muscle relaxation more rapidly, completely, and from a greater depth of blockade than neostigmine including after a rapid sequence intubation dose with rocuronium [3, 4]. In children, sugammadex reduces postoperative nausea and vomiting [5], may improve time to return of bowel function and reduce urinary retention in some populations [6], and shortens time to extubation, while reducing postoperative agitation [4, 7]. Finally, sugammadex availability may increase use and dosing of neuromuscular blocking medications, which may have implications for patient safety [8, 9]. For instance, use of neuromuscular blockade during intubation may reduce the incidence of laryngospasm or bronchospasm and increase the success of first pass at intubation [6, 10], and increased use of deep neuromuscular blockade during surgery may improve surgical conditions, enhancing patient safety [11].

However, concerns around sugammadex's cost may limit use [12]. Manufacturer label doses of 2 mg/kg or 4 mg/kg doses for pediatric patients are often far less than the standard 200 and 500 mg single use vials, thus significant medication waste may occur [12]. One proposed solution is to fractionate medication vials for pediatric patients to minimize waste. Therefore, beginning in 2021 C.S., Mott Children's Hospital undertook a quality improvement (QI) initiative in which vials of sugammadex were fractionated under a laminar airflow hood into 25, 50, or 100 mg aliquots. To understand the impact of this initiative on clinical practice, we examined how sugammadex dosing practices changed prior to and after the intervention. We hypothesized that following the intervention there would be a decrease in medication waste, improved adherence to recommended dosing guidelines particularly seen in a decrease in doses exceeding 4 mg/kg, and a decline in overall administered dose. We elected to focus on dosing of sugammadex rather than to perform a cost analysis as staffing, pharmacy, material, and other costs are subject to wide variability across institutions and time.

Our formal S.M.A.R.T. (specific, measurable, achievable, relevant, and time-bound) aim is as follows: (1) reduce sugammadex waste (2) Reduce sugammadex dose, and (3) improve compliance with published dosing guidelines in pediatric patients by fractionating full vials of sugammadex into 25 mg, 50 mg, and 100 mg aliquots that must be obtained in person from pharmacy staff who will distribute them according to patient's actual body weight.

Finally, we used what we learned from implementing this intervention to inform the development of a cognitive framework with which to anticipate unintended consequences of vial fractionation for sugammadex or other expensive medications (such as intravenous acetaminophen or remimazolam) [13, 14], which may lead to reductions in cost savings and increased risks to patients, that can be generalized across different populations and clinical settings.

## 2. Materials and Methods

### 2.1. Intervention Rationale and Description

**Figure 1:**
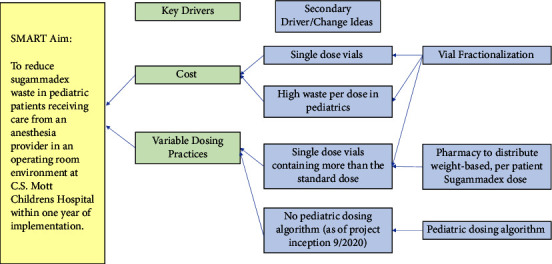
Weight-based standardized sugammadex dosing in pediatrics a Key Driver Diagram.

In September 2020, the Department of Anesthesiology undertook a QI initiative at C.S. Mott Children's Hospital in Ann Arbor, Michigan, to reduce sugammadex waste in pediatric patients receiving care from an anesthesia provider in an operating room environment. Key drivers motivating this initiative are described in Figure 1. The initiative utilized the Johns Hopkins Nursing Evidence-Based Practice Model [15] to provide a structured template for the appraisal, definition, and development of a solution for sugammadex medication waste and variable dose administration in pediatric patients undergoing anesthesia. We first identified stakeholders, which included anesthesiologists, nurse anesthetists, the lead operating room pharmacist, pharmacy personnel, an anesthesia manager, and the lead pharmacist. Preliminary data regarding current usage patterns for July 2019-July 2020 was reviewed by stakeholders assuming strict adherence to single patient per vial administration practice, in alignment with departmental policy. The analysis suggested that if vials were fractionated waste could be reduced by up to 43%, with a corresponding reduction in medication costs.

Based on this analysis the decision was made to proceed with sugammadex vial fractionation. First, a medication use algorithm was developed using train-of-four- (TOF-) based dosing in which we estimated a rounded dose for each patient weight in kg that would fall within 10% of 2 mg/kg (Supplement 1). Using predrawn syringes containing 25 mg, 50 mg, or 100 mg of sugammadex this algorithm resulted in an estimated average dose of 2.18/kg (lowest rounded dose: 1.79/kg, and the highest rounded dose: 3.33/kg). Second, pharmacy staffs were trained to distribute predrawn syringes of sugammadex that would allow for 2 mg/kg dosing (±10%). If a provider requested additional sugammadex, an additional 2 mg/kg would be provided. Full vials of sugammadex were made available in the in-operating room anesthesia carts to be used for emergency purposes, and when fractionated syringes were not available.

### 2.2. Data Source

To analyze changes in sugammadex dosing practices following the intervention, we obtained data from the perioperative research database at the University of Michigan. Institutional review board approval was obtained with a waiver of consent (University of Michigan, Ann Arbor, MI, HUM00230491), as this research was exempt research consisting of secondary analysis of a limited dataset.

### 2.3. Population

**Figure 2:**
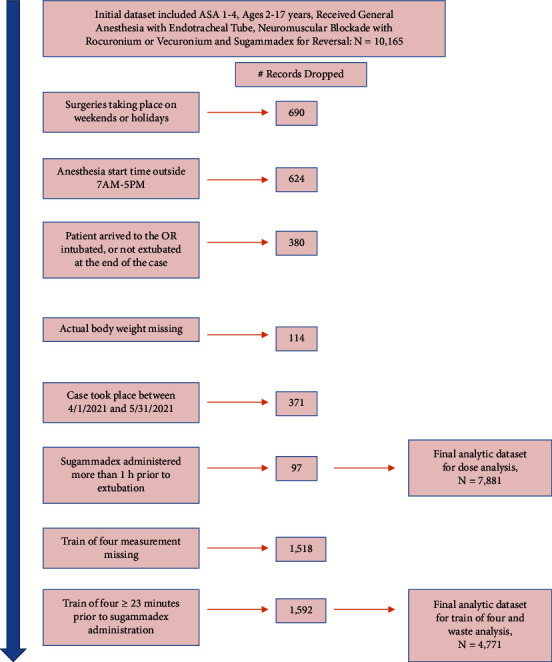
Inclusion/exclusion flowchart.

We included children ages 2–17 years undergoing general anesthesia with an endotracheal tube who received sugammadex, 1/1/2019 to 3/15/2023, excluding 4/1/2021−5/31/2021 during intervention implementation. Anesthetics taking place outside of 7 AM-5 PM, Monday–Friday, were excluded as the operating room pharmacy is closed. Additionally, in the analysis examining dosing guideline compliance, we excluded patients with no recorded train of four (TOF) measurement, and patients with TOF recorded more than 23 minutes prior to sugammadex administration, chosen because 75% of doses occurred within this timeframe.  Figure 2 illustrates the inclusion/exclusion flowchart.

### 2.4. Outcomes

Our outcome measures were: (1) administered sugammadex dose in mg/kg of actual body weight (ABW), (2) the percentage of cases receiving <1.8 mg/kg, 1.8–2.2 mg·kg, 2.2–3.6 mg/kg, 3.6–4.4 mg/kg and >4.4 mg/kg (2 mg/kg or 4 mg/kg product label dose of ±10%), and (3) an estimate of sugammadex waste per case in mg/kg before and after the intervention. To estimate sugammadex waste, we assumed that the minimum number of 200 mg or 500 mg sugammadex vials (preintervention) or 25, 50, or 100 mg predrawn syringes (postintervention) would be used for each case. For instance, if a patient weighing 20 kg with a TOF of 3 twitches received a 40 mg dose of sugammadex, prior to the intervention that would have resulted in 160 mg of waste (from a 200 mg vial) or 8 mg/kg, whereas after the intervention that would have resulted in 10 mg of waste (from a 50 mg syringe) or 0.14 mg/kg.

We also measured (4) concordance with manufacture label, by examining dose compliance accounting for actual body weight and documented TOF measurement. Per manufacturer's dosing guidelines, patients having 0 or 1 measured twitches should receive 4 mg/kg dosing (3.6–4.4 mg/kg) and patients with 2 or more measured twitches should receive 2 mg/kg dosing (1.8–2.2 mg/kg). In addition, since TOF measurement must be performed immediately prior to the time of sugammadex administration to effectively inform dosing decisions, we used the TOF measure taken closest to (but still prior to) sugammadex administration. Noting that some TOF measurements were taken long before sugammadex was administered, we examined the distribution of time between TOF measure and sugammadex administration and selected cases in which the time elapsed between TOF measurement and sugammadex administration fell within the shortest 75^th^ percentile of times, which corresponded to 23 minutes.

### 2.5. Exposures

The primary exposure was the date of sugammadex administration, and whether it occurred before 4/1/2021 vs. after 5/31/2021. We examined differences in important covariates including surgery type, the presence of one of twelve validated pediatric complex chronic conditions, comorbidities such as sleep apnea, and obesity, American Society of Anesthesiologists classification, whether the surgery was emergent, and patient gender and age group (2–5 years, 6–11 years, 12–17 years).

### 2.6. Statistical Analyses

We used absolute standardized differences (ASD) to describe differences in the patient cohort prior to and after the QI intervention was implemented. We evaluated whether adjusted analyses would be necessary based on the findings from this analysis, deciding a priori that we would consider ASD less than 0.2 to be nonsignificant differences in groups between the two time periods. We used percentages, histograms, scatterplots, and chi-squared tests to describe changes in dosing practices and waste prior to and after the intervention. We graphically illustrated changes in outcome measures prior to and after the QI intervention were implemented using run charts.

## 3. Results

A total of 7,889 cases were included (preintervention: 3,285; postintervention: 4,604). Patient and surgery characteristics pre- and postintervention were similar (Table 1).

### 3.1. Dose Quantity

**Figure 3:**
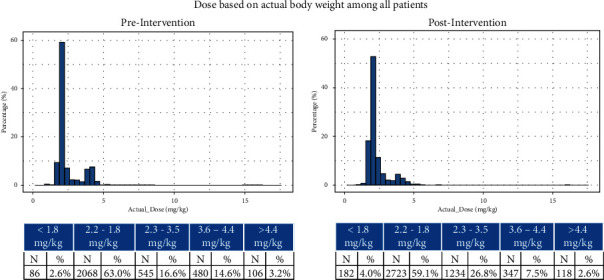
Sugammadex dose pre- and postintervention based on actual body weight only.

**Figure 4:**
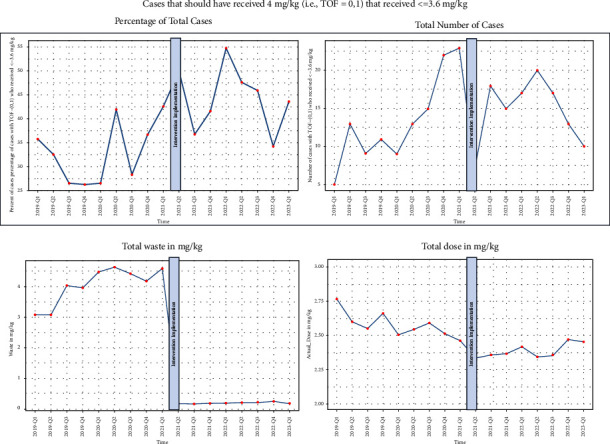
Run charts showing changes in parameters before and after quality improvement initiative.

Postintervention, mean dose was 2.4 mg/kg (SD: 0.96) compared to 2.5 mg/kg (SD: 1.3) preintervention; median dose was similar pre- and postintervention (2.1 (IQR: 2.0, 2.5) vs. 2.1 (IQR: 2.0, 2.4)) (Figures 3 and 4). Postintervention, smaller proportion of cases received standard 2 or 4 mg/kg doses of ±10% (77.6 vs. 66.7%); a larger proportion of cases received 2.2–3.6 mg/kg (16.6 vs. 26.8%), or <1.8 mg/kg (2.6 vs. 4.0%) (*p* < 0.0001). Fewer cases (3.2 vs. 2.6%) received >4.4 mg/kg.

### 3.2. Waste

Preintervention, mean waste per case was 4.2 mg/kg (SD: 4.1) compared to 0.22 mg/kg (SD: 0.38) postintervention and median waste was 2.5 mg/kg (IQR: 1.1, 6.6) compared with 0 mg/kg postintervention (IQR: 0.0, 0.3) (Figure 4).

### 3.3. Dose Compliance

A TOF value was documented for 6,289 cases, and within 23 mins (within the 75^th^ percentile of the time elapsed between TOF measurement and sugammadex administration) in 4,771 cases.

**Figure 5:**
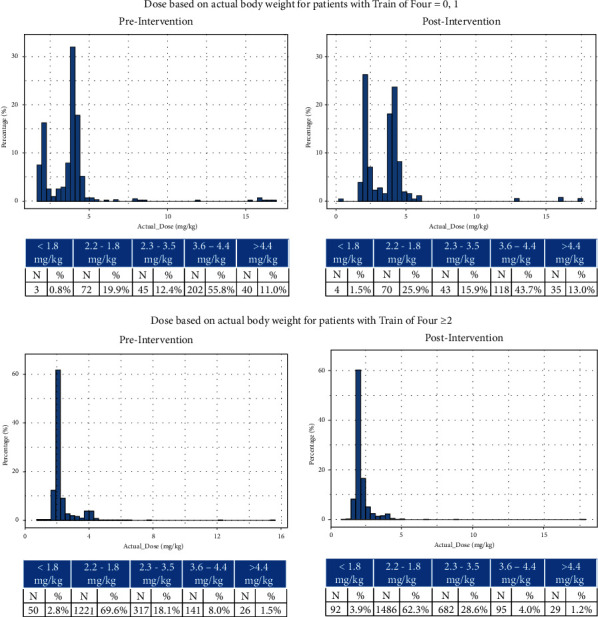
Sugammadex dose pre- and postintervention based on actual body weight and train of four measurement.

Among cases with 0-1 twitches, which should receive 4 mg/kg dosing (±10%), fewer cases received at least 3.6 mg/kg postintervention (56.7%) compared with preintervention (66.8%). Furthermore, a greater proportion received less than 2.2 mg/kg postintervention (27.4%) than preintervention (20.7%), and a greater proportion received between 2.2 and 3.6 mg/kg (21.9% vs. 13.7%) (Figures 4 and 5).

Among cases with 2 or more twitches, which should receive 2 mg/kg dosing, the proportion receiving <2.2 mg/kg declined from 72.4% to 66.2%. The proportion of patients receiving >3.6 mg/kg also declined from 9.5% to 5.2%. A higher proportion of patients received between 2.2 and 3.5 mg/kg after the intervention was implemented (18.1% vs. 28.6%).

## 4. Discussion

In this analysis of a QI intervention in which sugammadex syringes were fractionated into smaller quantities to reduce waste and improve compliance with dosing guidelines, we noted a reduction in sugammadex waste, but only minimal reductions in sugammadex dose. However, unexpectedly, compliance with labelled dosing guidelines also decreased. A greater proportion of patients with 0 or 1 measured twitches received less than the indicated 4 mg/kg (±10%), and even more concerningly, a larger proportion also received less than 2 mg/kg (±10%). This is important because underdosing of sugammadex can cause residual paralysis which could lead to pulmonary complications. Thus, while sugammadex vial fractionation could enable increased access to sugammadex and other cost prohibitive medications, it may introduce unintended consequences.

Though this analysis demonstrates that sugammadex waste decreased with associated per case cost savings, this analysis did not take into consideration changes in the population of patients which receive sugammadex. Past work has demonstrated that once an institution permits access to sugammadex, use increases as providers gain experience with the medication effects and begin using it in a broader population of patients [2, 8]. In addition, a recent study found that dosing of sugammadex-compatible neuromuscular blocking medications increased after the introduction of sugammadex to the institution, which could also necessitate increased sugammadex dosing [16]. Failure to account for unintended consequences such as changes in use patterns of sugammadex and neuromuscular blocking agents due to increased availability of sugammadex could lead to cost savings which are ultimately not actualized or less than expected.

In light of this data, we developed a framework for measuring the associated costs and benefits associated with sugammadex vial fractionation. We have paid particular attention to estimating the unintended consequences that may arise with sugammadex vial fractionation, which may lead to reductions in cost savings and increased risks to patients as well as benefits, that can be generalized across different populations and clinical settings. Components must be individualized to specific institutional environments accounting for patient population served and standard operating room and pharmacy workflow.

1. Will the QI initiative cause a change in workflow for obtaining sugammadex? In this case, sugammadex was only available at the pharmacy, necessitating a conversation with a pharmacist or pharmacy technician each time the medication was obtained. This workflow, combined with routine dispensing of 2 mg/kg doses, may discourage anesthesia providers from giving larger doses even if needed because an extra trip to the pharmacy would be required. At our institution, retrieval of additional medication from a dedicated pharmacy within the operation room suite is feasible within five minutes. Despite these low barriers, we still saw decreased compliance with dosing guidelines. Other institutions with different workflow and/or pharmacy locations and protocols may need to account for associated costs when considering implementing this intervention locally.
2. Does the way in which the vials are fractionated inadvertently incentivize lack of compliance with dosing guidelines? In retrospect, examining the dose quantities of the fractionated syringes created in this QI initiative, it is easy to see how a large number of patients would end up receiving between 2 mg/kg and 4 mg/kg because many providers will administer the entire syringe rather than exactly 2 mg/kg if there is a small quantity left over. This is only problematic insofar as waste reduction is not maximized. However, in this initiative, doses that were as much as 10% less than 2 mg/kg were also considered acceptable for patients with two or more twitches. Therefore, it is unsurprising that an increased proportion of patients received less than the recommended 2 mg/kg. Recent evidence has shown that residual paralysis may be present in up to 13% of patients receiving the dose recommended by the manufacturer based on measured train of four count; therefore, underdosing may be more clinically important than previously thought [16]. Attentive monitoring of residual paralysis with routine quantitative neuromonitoring could be a way to mitigate this risk, however, with its own associated costs.
3. Evidence suggests that when sugammadex is introduced to an institution, the proportion of patients who receive it and dosing of sugammadex-compatible neuromuscular blocking medications increases, likely due to the availability of more reliable reversal [2, 8, 16]. These increases in use may reduce cost savings as more patients receive this costly medication. This may lead to unanticipated clinical benefits (e.g., fewer complications during intubation), or costs (e.g., increased rate of residual paralysis during recovery) depending on how the intervention is implemented. Organizations must decide whether policies will be put into place to limit the population of patients receiving sugammadex or whether the benefits of the medication are sufficient to justify its increased use.
4. A recent survey suggested that pediatric anesthesia providers monitor train of four less carefully when sugammadex is available [17]. This, combined with neuromuscular blocking medication dose escalation could lead to increased rates of residual paralysis and avoidable pulmonary complications. Pharmacy and operating room procedures implemented to reduce access to sugammadex may further increase these risks by increasing the likelihood of underdose. Reinforcement of and incentivizing appropriate train of four monitoring or quantitative neuromonitoring to monitor depth of neuromuscular blockade may help mitigate many negative unintended consequences.
5. Finally, implementation of this QI initiative may introduce new material and environmental costs, depending on how it is implemented. These could include the use of additional syringes and needles, increased use of supplies needed for qualitative or quantitative neuromonitoring, increased use of other medications such as neuromuscular blocking agents, or increased use of endotracheal intubation with costs associated with that, as well as the costs associated with disposing of these items. These costs may be difficult to anticipate, and are likely to vary across institutions and time; thus, must be anticipated, measured, and addressed on an individual institution basis.

This study has several limitations: First, it was conducted at a single tertiary care pediatric hospital, limiting generalizability. We have elected to focus on the unintended consequences on sugammadex dosing, which are likely to be generalizable to other institutions implementing a vial fractionation strategy, rather than specific associated costs associated with the QI initiative which are more likely to be dependent on institution-specific staffing and processes and thus less generalizable. Second, TOF data were missing in 20% of cases, limiting our ability to include numerous patients in an important part of the analysis. Third, this analysis does not take into account whether more cases received sugammadex after the intervention than prior to it; however, recent work has suggested that this is not only common, but that neuromuscular blocking medication dose escalation may also occur when sugammadex is made available [1, 2, 9]. Fourth, no data are available on patient outcomes such as residual neuromuscular blockade or respiratory complications that may have resulted from sugammadex underdosing. Fifth, estimates of waste reduction are projected and may not reflect actual waste; as noted, a smaller number of fractionated syringes were created than were required each day to minimize waste; therefore, patients receiving sugammadex later in the day likely received a full vial, reducing the actual amount of waste saved. Sixth, our study does not consider changes in dosing and waste that may have occurred in patients less than 2 years of age, in whom sugammadex remains off label. And finally, recent work suggests that manufacture label guidance may not correspond to the dose needed for complete neuromuscular recovery; residual paralysis may be present in up to 13% of patients receiving the dose recommended by the manufacturer based on measured train of four counts [16]. Implementation of quantitative neuromonitoring may mitigate some of these risks, but it requires the purchase and use of specialized equipment and disposables and clinician education.

In conclusion, a QI initiative to reduce sugammadex dosing and waste was successful in its primary goal of reducing medication waste; mean and median dosing did not appreciably change. However, compliance with labelled dosing recommendations decreased, resulting in a larger proportion of patients with deep paralysis receiving smaller than recommended doses. Furthermore, hidden costs such as increased sugammadex use, time costs to staff, pharmacy costs, and material costs may ultimately reduce the benefits of such a program and must be addressed individually within each institution. We have provided a framework which other institutions can use to consider potential unintended consequences, which can be used alongside institution-specific data on staffing, material, and other costs, in determining whether dose fractionation of sugammadex makes sense for their institution.

## Figures and Tables

**Table 1:** Patient and surgery characteristics before and after intervention.

Variable	Full sample (*n* = 7889)		Preintervention (*n* = 3285)		Postintervention (*n* = 4604)		Absolute standardized difference
	*N*	%	*N*	%	*N*	%	
Primary surgery type							
Burns	18	0.01	13	0.40	5	0.11	0.174
Lower arm and hand	164	0.06	54	1.64	110	2.39	
Head	1908	0.74	767	23.35	1141	24.78	
Intrathoracic	840	0.32	369	11.23	471	10.23	
Knee, popliteal	283	0.11	121	3.68	162	3.52	
Lower abdominal	1060	0.41	502	15.28	558	12.12	
Lower leg	258	0.10	95	2.89	163	3.54	
Neck	308	0.12	144	4.38	164	3.56	
Obstetric	3	0.00	1	0.03	2	0.04	
Pelvis	101	0.04	39	1.19	62	1.35	
Perineum	272	0.11	108	3.29	164	3.56	
Radiology	850	0.33	300	9.13	550	11.95	
Shoulder	104	0.04	46	1.40	58	1.26	
Spine	105	0.04	45	1.37	60	1.30	
Thoracic	241	0.09	92	2.80	149	3.24	
Upper abdomen	853	0.33	376	11.45	477	10.36	
Upper arm and elbow	199	0.08	91	2.77	108	2.35	
Upper leg	247	0.10	100	3.04	147	3.19	
Feudtner complex chronic conditions							
Neurologic/neuromuscular disease	686	0.27	296	9.01	390	8.47	0.019
Cardiovascular disease	1983	0.77	845	25.72	1138	24.72	0.023
Respiratory disease	320	0.12	142	4.32	178	3.87	0.023
Renal/urologic disease	464	0.18	199	6.06	265	5.76	0.013
Gastrointestinal	1213	0.47	511	15.56	702	15.25	0.008
Hematologic/immunologic	770	0.30	278	8.46	492	10.69	0.076
Metabolic	811	0.31	338	10.29	473	10.27	0
Congenital/genetic	1086	0.42	477	14.52	609	13.23	0.037
Malignancy	897	0.35	387	11.78	510	11.08	0.022
Diseases of prematurity	20	0.01	9	0.27	11	0.24	0.007
Transplant	266	0.10	118	3.59	148	3.21	0.021
Requires a medical device	1325	0.51	571	17.38	754	16.38	0.027
Sleep apnea	790	0.31	342	10.41	448	9.73	0.023
Obesity	1468	0.57	635	19.33	833	18.09	0.035
Underweight	356	0.14	134	4.08	222	4.82	0.041
ASA classification							
ASA class 1	2237	0.86	912	27.76	1325	28.78	0.026
ASA class 2	2869	1.11	1195	36.38	1674	36.36	
ASA class 3	2580	1.00	1090	33.18	1490	32.36	
ASA class 4	203	0.08	88	2.68	115	2.50	
Emergency surgery	764	0.30	259	7.88	505	10.97	0.106
Female sex	3458	1.34	1413	43.01	2045	44.42	0.028
Age group							
12–17 years	3741	1.45	1586	48.28	2155	46.81	0.031
6–11 years	2261	0.87	932	28.37	1329	28.87	
2–5 years	1887	0.73	767	23.35	1120	24.33	

## Data Availability

The data used to support the findings of this study may be released upon application to the University of Michigan Department of Anesthesiology Clinical Research Committee or who can be contacted at anes-acrc@med.umich.edu.

## Additional Points

*Clinical Implications*. Fractionating vials of sugammadex into smaller aliquots for pediatric patients may reduce waste but may introduce unintended consequences, such as medication underdosing, depending on the institutional protocol for obtaining sugammadex, constituting a potential threat to patient safety. Other unintended consequences that reduce potential cost savings may also occur which may negatively impact any cost savings.

## Supplementary Materials

Supplementary Materials Supplemental Table 1: rounded dose algorithm for fractionated sugammadex vials.
