# Supplementary figures and images for: Weight-Based Standardized Sugammadex Dosing in Pediatrics: A Quality Improvement Initiative to Improve Compliance with Dosing Guidelines and Reduce Waste and Cost
Source: Anesthesiol Res Pract. 2024 Aug 24;2024:6049114. doi: 10.1155/2024/6049114 (PMC11366054; doi:10.1155/2024/6049114)

**Supplemental Table 1. Rounded Dose Algorithm for Fractionated Sugammadex Vials**


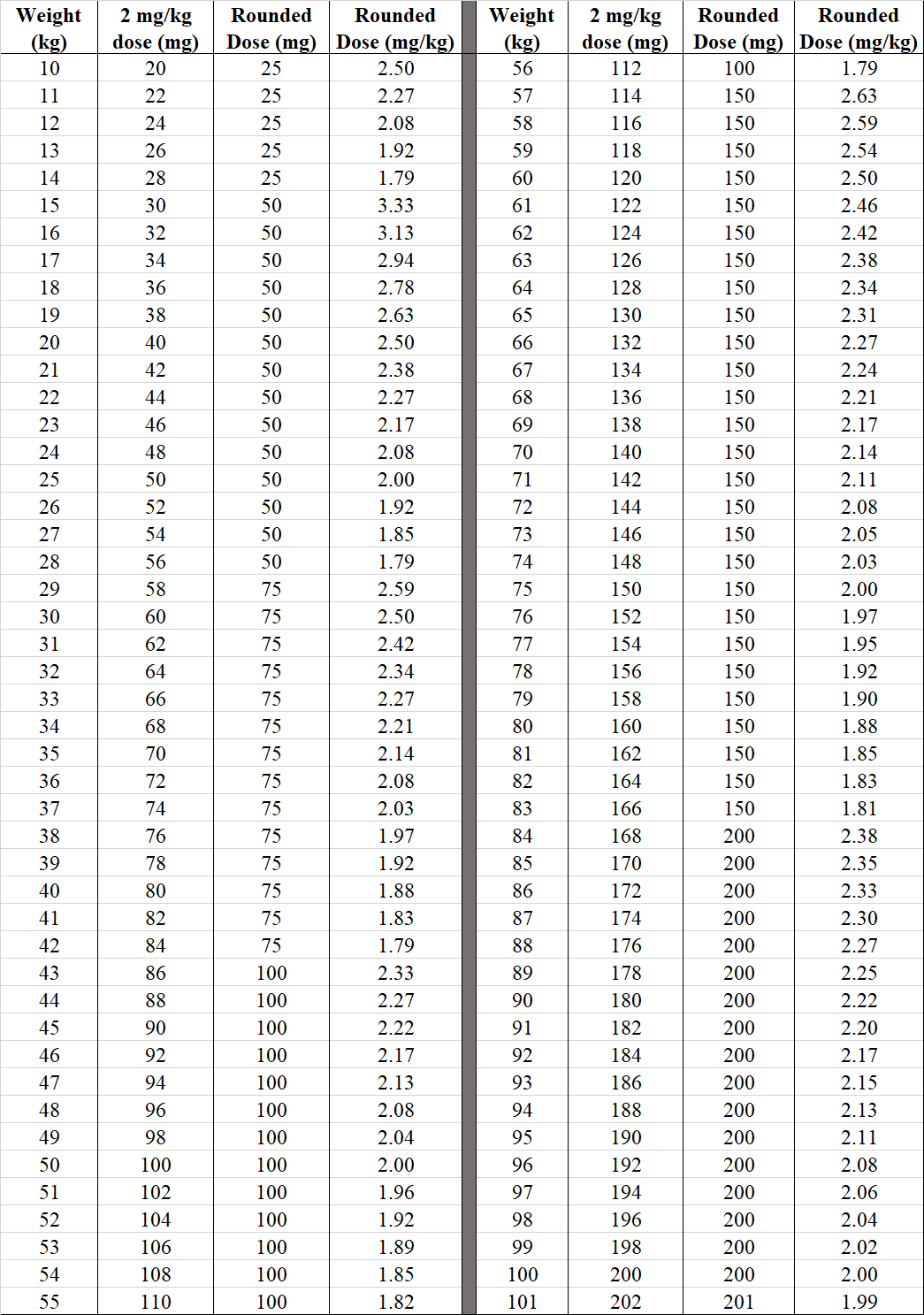

Supplement: Supplementary Materials — Supplemental Table 1: rounded dose algorithm for fractionated sugammadex vials. [file 6049114.f1.docx]
